# Genome-wide identification and gene expression analysis of the 14–3-3 gene family in potato (Solanum tuberosum L.)

**DOI:** 10.1186/s12864-022-09037-y

**Published:** 2022-12-07

**Authors:** Feiyan He, Shaoguang Duan, Yinqiao Jian, Jianfei Xu, Jun Hu, Zhicheng Zhang, Tuanrong Lin, Feng Cheng, Guangcun Li

**Affiliations:** 1 Key Laboratory of Biology and Genetic Improvement of Tuber and Root Crop, Institute of Vegetables and Flowers, Chinese Academy of Agricultural Sciences, Ministry of Agriculture and Rural Affairs, Beijing, 100081 China; 2 Wulanchabu Academy of Agricultural and Forest Sciences, Wulanchabu, Inner Mongolia, 012000 China

**Keywords:** Potato, *14–3-3* gene family, Genome-wide identification, Expression analysis, Abiotic stress

## Abstract

**Background:**

14–3-3 proteins are essential in regulating various biological processes and abiotic stress responses in plants. Although 14–3-3 proteins have been studied in model plants such as *Arabidopsis thaliana* and *Oryza sativa*, there is a lack of research on the *14–3-3* gene family in potatoes (*Solanum tuberosum* L.).

**Results:**

A total of 18 *14–3-3* genes encoding proteins containing a typical conserved PF00244 domain were identified by genome-wide analysis in potatoes. The *St14–3-3* gene family members were unevenly distributed across the chromosomes, and gene structure analysis showed that gene length and intron number varied greatly among the members. Phylogenetic analysis of 14–3-3 proteins in potatoes and other plant species showed that they could be divided into two distinct groups (ε and non-ε). Members in the ε group tended to have similar exon-intron structures and conserved motif patterns. Promoter sequence analysis showed that the *St14–3-3* gene promoters contained multiple hormone-, stress-, and light-responsive cis-regulatory elements. Synteny analysis suggested that segmental duplication events contributed to the expansion of the *St14–3-3* gene family in potatoes. The observed syntenic relationships between some *14–3-3* genes from potato, *Arabidopsis*, and tomato suggest that they evolved from a common ancestor. RNA-seq data showed that *St14–3-3* genes were expressed in all tissues of potatoes but that their expression patterns were different. qRT-PCR assays revealed that the expression levels of nearly all tested *St14–3-3* genes were affected by drought, salt, and low-temperature stresses and that different *St14–3-3* genes had different responses to these stresses.

**Conclusions:**

In summary, genome-wide identification, evolutionary, and expression analyses of the *14–3-3* gene family in potato were conducted. These results provide important information for further studies on the function and regulation of *St14–3-3* gene family members in potatoes.

**Supplementary Information:**

The online version contains supplementary material available at 10.1186/s12864-022-09037-y.

## Background

14–3-3 proteins are a class of highly conserved, broadly expressed regulatory proteins that are present in virtually every eukaryotic organism [1, 2]. 14–3-3 proteins were first discovered in bovine brain tissue by Moore and Perez (1967) and later named 14–3-3 proteins according to their electrophoretic mobility [3, 4]. In plants, 14–3-3 proteins can form homodimers or heterodimers. Because they are part of the G-box protein complex, they are also named G-box regulatory factor or general regulatory factor (GRF) or G-box factor 14–3-3 homolog (GF14) proteins [5, 6]. The 14–3-3 protein family was first widely studied in animal cells, and the first function identified was the activation of the synthesis of neurotransmitters [7]. It was later discovered that family members had neuroprotective effects [8–10]. Since 1999, 14–3-3 proteins have been widely studied in *Arabidopsis thaliana*, *Oryza sativa* (rice), *Zea mays* (maize), and other model plants, where they have been found to play important roles in processes such as cell division, growth, development, metabolism, and resistance to stress [11].

In plants, the 14–3-3 protein family can be divided into the epsilon (ε) and non-epsilon (non-ε) classes according to protein sequence similarity and the number of introns. The ε class isoforms (ε, μ, π, ρ, σ) have more exons than non-ε class isoforms [12]. Although members of the 14–3-3 protein family have similar structures, they bind to different ligands, perform different functions, and function in different tissues. 14–3-3 proteins are phosphoserine-binding proteins that regulate the activity of multiple targets through direct protein-protein interactions [13]. They exert their functions mainly by regulating ion channels and hormone signaling pathways involved in growth, development, and response to abiotic stress [14]. For example, 14–3-3 proteins regulate ion channels in plant cells by interacting with H^+^-ATPase. In sugar beet, the abundance of 14–3-3 proteins in the plasma membrane increases under cold or osmotic stress, and this increase is associated with increased H^+^-ATPase activity [13]. In addition, 14–3-3 proteins can also regulate K^+^ channels; the *Solanum lycopersicum* (tomato) 14–3-3 proteins TFT4 and TFT7 can stimulate the K^+^ channel to transition from a dormant state to an activated state, thereby changing the membrane potential [15]. 14–3-3 proteins also play a role in the abscisic acid (ABA) signaling pathway, a major hormone signal transduction pathway whose main physiological function is to regulate stomatal opening, maintain cell osmotic balance, and prevent plant water loss under stress conditions such as drought or salinity. For example, in *Vicia faba*, an increase in ABA under drought stress allows 14–3-3 proteins to bind to proteins in guard cells, leading to stomatal closure, thereby improving drought tolerance [16].

In recent years, a variety of *14–3-3* gene family members have been identified in many plants. *14–3-3* genes have been demonstrated to play crucial roles in stress response, and functional analysis revealed that numerous *14–3-3* genes could confer tolerance to single or multiple stresses in transgenic plants [17]. For example, overexpression of *Arabidopsis GRF9* enhances plant drought tolerance, while deletion of this gene causes poor root development and weak growth [18]. Overexpression of *Triticum aestivum TaGF14b* confers drought and salt tolerance to transgenic tobacco plants through the ABA signaling pathway [19]. Overexpression of *BdGF14a* and *BdGF14d* from *Brachypodium distachyon* was found to enhance the drought and salt tolerance of transgenic plants, respectively [20, 21]. In addition, some *14–3-3* genes have been found to influence stress tolerance negatively. For instance, RARE COLD INDUCIBLE 1 A functions as a negative regulator of cold and freezing stress tolerance in *Arabidopsis*, and knockout of *AtGF14ψ* (*AtGRF3*) improves tolerance to cold stress in *Arabidopsis* [22], indicating that it also functions as a negative regulator. Rice *osgf14b* mutants showed higher resistance to drought and osmotic stress than the wild type, whereas *OsGF14b*-overexpressing rice plants displayed higher sensitivity to stress, revealing the negative role of *OsGF14b* in osmotic and drought resistance [23]. Overexpression of *Glycine soja GF14o* in *Arabidopsis* also resulted in lower tolerance to drought stress with down-regulated expression of stress-responsive genes, suggesting that it acts as a negative regulator of drought tolerance [24]. Recently, an increasing number of gene expression studies have provided evidence that 14–3-3 s in plants may function under multiple stresses [13]. The expression of most *GRF* genes in rice was found to change under heat, cold, and salt stresses [25]. *ZmGF14–6* of maize (which encodes a 14–3-3 protein) is upregulated in response to fungal infection and salt treatment but downregulated in response to drought stress [26]. In addition, the expression levels of the 14–3-3 protein-encoding gene *MdGRF11* in apple increase significantly under salt and low-temperature stress [27].

Potato (*Solanum tuberosum* L.), which belongs to the Solanaceae family, is the fourth largest food crop in the world [28]. It has 12 chromosomes with a medium size genome of approximately 840 Mb [29]. Potatoes are grown worldwide, and they are extremely vulnerable to various abiotic and biotic stresses such as chilling, drought, salt, pests, and pathogens, which cause significant production losses worldwide. Increasing the resistance of potatoes to biotic/abiotic stresses to increase yield production is a hot research topic, and mining stress resistance genes at the genetic level is of great significance for potato germplasm utilization and variety improvement. The availability of the complete genome sequence of potatoes creates an opportunity to explore stress-responsive gene families that could provide tolerance against environmental stresses. Therefore, we conducted a detailed comparative genome-wide analysis of the *14–3-3* gene family in potatoes, identifying 18 *14–3-3* genes. We conducted comprehensive analyses of gene structure, phylogenetic relationship, promoter elements, chromosome distribution, and expression pattern. Our results suggest that potato *14–3-3* gene family members play various roles in development and stress response.

## Results

### Genome-wide identification and characterization of *14–3-3* family genes in potato

A total of 18 *14–3-3* genes were identified in the potato genome; these genes were named *StGRF1–18* based on their physical locations on the chromosomes (Table 1). The lengths of the coding sequences of StGRFs ranged from 576 bp (*StGRF4*) to 840 bp (*StGRF11*), and the encoded protein lengths ranged from 191 to 279 amino acids. The MWs ranged from 21.72 kDa (StGRF4) to 31.34 kDa (StGRF11), and the PI values ranged from 4.62 (StGRF17) to 5.63 (StGRF6). The GRAVY values of StGRF proteins ranged from − 0.649 (StGRF7) to − 0.196 (StGRF4), suggesting that StGRF proteins are hydrophilic. Subcellular localization prediction revealed that 14–3-3 proteins of potatoes are localized in the plasma membrane, cytoplasm, nucleus-plasma membrane, chloroplast, and mitochondria. Interestingly, 44.4% of StGRFs are predicted to be located on the plasma membrane, and 22.2% are predicted to localize to the nucleus-plasma membrane.

**Table 1:** List of all StGRF genes identified in the potato genome

Name	Gene ID	Chr.	Genomic localization	CDS	Exon	AA	MW (kDa)	PI	GRAVY	Subcellular localization	Subgroup
*StGRF1*	Soltu.DM.02G006890.1	2	20,885,062–20,878,027	783	4	260	29.31	4.66	−0.517	plasma membrane	non-ε
*StGRF2*	Soltu.DM.03G004400.1	3	4,634,852–4,630,049	777	4	258	29.22	4.67	−0.505	plasma membrane	non-ε
*StGRF3*	Soltu.DM.03G004400.2	3	4,634,852–4,630,049	783	3	260	29.35	5.04	−0.310	plasma membrane	non-ε
*StGRF4*	Soltu.DM.03G004400.3	3	4,634,852–4,630,049	576	1	191	21.72	5.53	−0.196	cytoplasm	non-ε
*StGRF5*	Soltu.DM.04G008400.1	4	8,744,749–8,743,192	768	4	255	28.77	4.70	−0.507	nucleus-plasma membrane	non-ε
*StGRF6*	Soltu.DM.04G008400.2	4	8,744,749–8,743,192	636	2	211	23.91	5.63	−0.340	plasma membrane	non-ε
*StGRF7*	Soltu.DM.04G029780.1	4	61,131,123–61,127,099	759	7	252	28.81	4.96	− 0.649	mitochondrion	ε
*StGRF8*	Soltu.DM.04G029780.2	4	61,131,123–61,127,099	756	7	251	28.73	4.93	−0.500	plasma membrane	ε
*StGRF9*	Soltu.DM.04G030120.1	4	61,487,551–61,489,550	780	4	259	29.31	4.78	−0.554	plasma membrane	non-ε
*StGRF10*	Soltu.DM.04G030690.1	4	62,155,349–62,152,023	759	4	252	28.62	4.80	−0.348	plasma membrane	non-ε
*StGRF11*	Soltu.DM.05G000660.1	5	529,878–533,438	840	7	279	31.34	5.18	−0.592	nucleus-plasma membrane	ε
*StGRF12*	Soltu.DM.07G020680.1	7	51,144,846–51,140,272	786	7	261	29.47	4.74	−0.552	chloroplast	ε
*StGRF13*	Soltu.DM.11G003170.1	11	3,178,138–3,182,867	750	4	249	28.20	4.76	−0.253	chloroplast	non-ε
*StGRF14*	Soltu.DM.11G003450.1	11	3,430,567–3,428,946	777	4	258	28.99	4.72	−0.493	plasma membrane	non-ε
*StGRF15*	Soltu.DM.12G006890.1	12	5,778,347–5,775,875	765	4	254	28.83	4.71	−0.562	nucleus-plasma membrane	non-ε
*StGRF16*	Soltu.DM.12G006890.2	12	5,778,306–5,775,875	747	3	248	28.09	4.89	−0.341	nucleus-plasma membrane	non-ε
*StGRF17*	Soltu.DM.12G028450.1	12	58,150,303–58,147,128	780	7	259	29.45	4.62	−0.541	cytoplasm	ε
*StGRF18*	Soltu.DM.12G028450.2	12	58,150,247–58,147,315	777	7	258	29.32	4.64	−0.530	cytoplasm	ε

Because of the large MWs of the proteins, different peptide chains can form different secondary structures. The secondary structure of a protein mainly includes α-helices, β-folds, β-turns, and random coils. The St14–3-3 proteins had similar secondary structures, indicating that these proteins may form similar higher-order structures and perform similar functions. The proportion of α-helices was the highest (64.87–71.77%), followed by random coils (18.55–26.88%), while the proportions of extended strands and β-turns were the lowest. β-turns are common stable secondary structures in polypeptide chains that mainly connect α-helices and β-folds in proteins. Antibody recognition, phosphorylation, glycosylation, and hydroxylation sites of proteins have been discovered to appear in β-turns frequently. StGRf14 consisted of 3.49% β-turns, and StGRF17 consisted of 0.77% β-turns (Table 2). The tertiary structure of a protein is formed by further winding and folding based on the secondary structure. It is mainly maintained by hydrophobic interactions between amino acid side chains, hydrogen bonds, and electrostatic interactions. Comparisons of predicted tertiary structures showed that the St14–3-3 proteins had similar three-dimensional conformations and were composed mostly of α-helices.

**Table 2:** The secondary structures of all *StGRF* genes identified in the potato genome

Gene name	Gene ID	α-helices	β-turns	Random coil	Extended strands
*StGRF1*	Soltu.DM.02G006890.1	68.85%	1.15%	23.46%	6.54%
*StGRF2*	Soltu.DM.03G004400.1	69.77%	2.33%	20.93%	6.98%
*StGRF3*	Soltu.DM.03G004400.2	68.46%	1.15%	21.92%	8.46%
*StGRF4*	Soltu.DM.03G004400.3	68.59%	1.05%	22.51%	7.85%
*StGRF5*	Soltu.DM.04G008400.1	68.24%	0.78%	23.92%	7.06%
*StGRF6*	Soltu.DM.04G008400.2	71.09%	1.42%	19.91%	7.58%
*StGRF7*	Soltu.DM.04G029780.1	67.46%	1.19%	23.81%	7.54%
*StGRF8*	Soltu.DM.04G029780.2	69.72%	1.59%	19.52%	9.16%
*StGRF9*	Soltu.DM.04G030120.1	70.27%	1.16%	21.62%	6.95%
*StGRF10*	Soltu.DM.04G030690.1	67.86%	1.19%	23.41%	7.54%
*StGRF11*	Soltu.DM.05G000660.1	64.87%	1.08%	26.88%	7.17%
*StGRF12*	Soltu.DM.07G020680.1	66.28%	1.53%	24.90%	7.28%
*StGRF13*	Soltu.DM.11G003170.1	69.48%	0.80%	21.69%	8.03%
*StGRF14*	Soltu.DM.11G003450.1	68.99%	3.49%	20.93%	6.59%
*StGRF15*	Soltu.DM.12G006890.1	67.32%	1.57%	24.41%	6.69%
*StGRF16*	Soltu.DM.12G006890.2	71.77%	1.21%	18.55%	8.47%
*StGRF17*	Soltu.DM.12G028450.1	70.66%	0.77%	21.62%	6.95%
*StGRF18*	Soltu.DM.12G028450.2	68.99%	1.55%	21.32%	8.14%

### Gene structure and conserved motif analysis of *14–3-3* family genes in potato

**Fig. 1:**
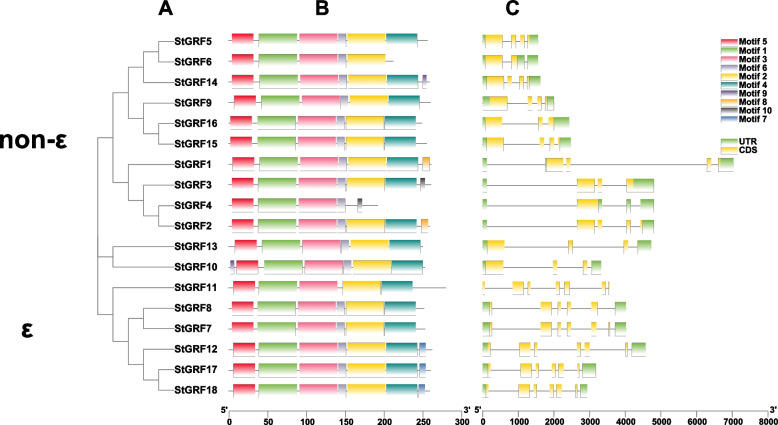
Phylogenetic relationships, gene structure, and conserved motifs of the StGRFs. **A** Construction of a rootless neighbor-joining phylogenetic tree comprising 18 StGRFs. **B** Distribution of conserved motifs within the StGRF proteins. The differently colored boxes represent different bases, and the motif numbers of the genes are shown in the colored boxes. **C** Exon/intron structures of *StGRF* genes. The yellow boxes represent exons, and the black lines represent introns. The lengths of the exons can be inferred from the scale at the bottom

Based on a phylogenetic tree of StGRF proteins, 12 StGRFs (StGRF1/2/3/4/5/6/9/10/13/14/15/16) belonged to the non-ε group, and 6 StGRFs (StGRF7/8/11/12/17/18) belonged to the ε group. The exon/intron patterns of the *StGRF* genes differed between the ε and non-ε groups, reflecting the divergence of *StGRF* genes during evolution (Fig. 1 A). To further characterize the StGRF proteins, we identified their conserved motifs using the MEME tool. A total of 10 conserved motifs were predicted, among which motif 1, motif 3, and motif 5 were conserved in all ε class and non-ε class StGRF proteins. Interestingly, all StGRF proteins contained motif 6, except for StGRF11, and all StGRF proteins contained motif 2 and motif 4, except for StGRF4 and StGRF6, respectively. The C-terminal motif, which may be responsible for the differences in target proteins, showed high variability among the members of the two subfamilies. In addition, motifs 8, 9, and 10 were mainly specific to the non-ε class StGRF proteins. In contrast, motif 7 was specific to the ε class proteins (Fig. 1 B). Exon/intron divergence plays a crucial role during evolution. The exon-intron structures of the *StGRF* genes were obtained by comparing the genomic and coding sequences. All *StGRF* genes in the ε group had six introns, while *StGRFs* in the non-ε group harbored 0–3 introns (Table 1; Fig. 1 C). The classification of StGRFs according to motif analysis was consistent with that obtained from phylogenetic analysis.

### Multiple sequence alignment and phylogenetic analysis of 14–3-3 family proteins in potato

**Fig. 2:**
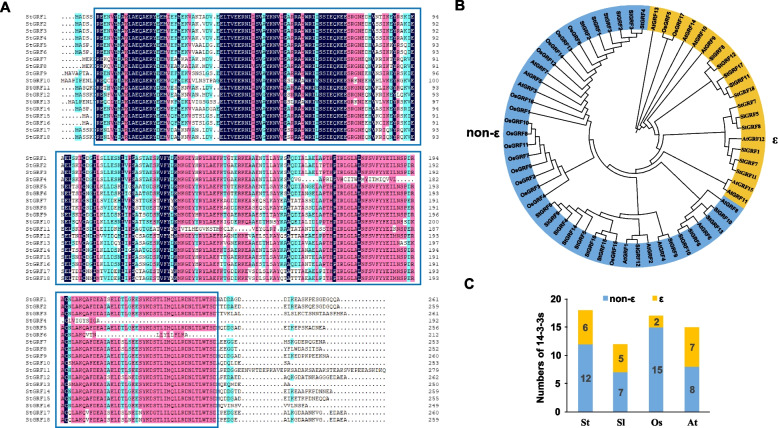
Multiple sequence alignment and phylogenetic tree analysis of StGRF proteins. **A** Multiple alignments of the full-length StGRF protein sequences. Amino acid residues identical in all eighteen sequences are shaded in light blue and pink, while highly conserved residues are shaded in navy blue. The typical conserved PFAM00244 domain is marked by the blue box. **B** Phylogenetic analysis of GRFs from *Arabidopsis*, rice, tomato, and potato. The neighbor-joining phylogenetic tree was constructed using Clustal X 2.0 and MEGA 6.0 with 1000 bootstrap replicates, and proteins in the ε and non-ε groups are shaded in gold and azure, respectively. **C** A visual display of counts for GRF members from potato (*St*), tomato (*Sl*), rice (*Os*), and *Arabidopsis* (*At*)

Multiple sequence alignment of the St14–3-3 protein family members revealed a high degree of similarity. The similarities among StGRF proteins ranged from 59.27% (StGRF11 and StGRF13) to 99.62% (StGRF17 and StGRF18) (Additional file 2), suggesting that they share relatively high levels of sequence similarity. Although the structures of the proteins in this family are highly conserved, the N-terminus and C-terminus, which form the core structures responsible for 14–3-3 protein functions, varied considerably according to multiple sequence alignment and analysis of conserved motifs (Fig. 1 B and Fig. 2 A). To explore the evolutionary relationships of the St14–3-3 proteins, phylogenetic trees were constructed using the amino acid sequences of St14–3-3, *A. thaliana* 14–3-3 (AtGRF), *O. sativa* 14–3-3 (OsGRF), and *S. lycopersicum* 14–3-3 (SlGRF) proteins. The StGRF proteins and the *Arabidopsis* and rice GRF proteins were located on different branches, indicating that they were not closely related. However, the StGRF proteins were more closely related to the tomato GRF proteins, suggesting that GRF proteins were conserved during the evolution of *Solanaceae* plant lineages. Similar to the *Arabidopsis* and rice *GRF* gene families, the *StGRF* gene family could be divided into ε and non-ε groups (Fig. 2 B). In addition, there were more non-ε group GRFs than ε group GRFs in *Arabidopsis*, rice, tomato, and potato (Fig. 2 C).

### Analysis of cis-elements in the promoter regions of *14–3-3* family genes in potato

**Fig. 3:**
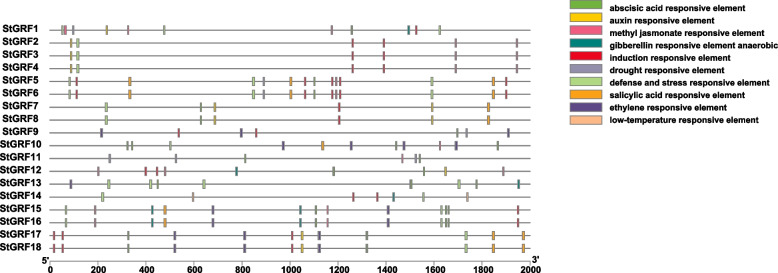
Visualization of the cis-regulatory elements within the promoters of *StGRF* gene family members. Six types of elements respond to hormones: abscisic acid responsive elements, auxin responsive elements, methyl jasmonate responsive elements, gibberellin responsive elements, ethylene responsive elements, and salicylic acid responsive elements. Four types of elements responsive to external environmental stresses: anaerobic induction elements, low-temperature responsive elements, drought responsive elements, and defense-and stress-responsive elements

Cis-elements are involved in transcriptional regulation and can respond to various stresses. To understand the expression and regulatory characteristics of the *St14–3-3* gene family members, we analyzed the promoter sequence of each of these genes (the 2 kb region upstream of the coding region) using the PlantCARE database (Fig. 3; Table 3). The results showed that hormone-and stress-related cis-elements were abundant in the promoter regions of *StGRF* genes. Six types of cis-elements (total of nine elements) associated with responses to different hormones were identified, namely the abscisic acid responsive element (ABRE), auxin responsive elements (AuxRR-core and TGA-element), methyl jasmonate (MeJA) responsive element (CGTCA-motif), gibberellin responsive elements (P-box and TATC-box), ethylene responsive element (ERE), and salicylic acid (SA) responsive element (TCA-element). *StGRF1*, *StGRF12*, and *StGRF13* contained both types of gibberellin-responsive elements. Auxin-responsive elements were found in *StGRF1*, *StGRF2*, *StGRF3*, *StGRF4*, *StGRF7*, *StGRF8*, *StGRF12*, *StGRF17*, and *StGRF18*. An ERE was present in most *StGRF* genes, namely *StGRF1*, *StGRF9*, *StGRF10*, *StGRF13*, *StGRF15*, *StGRF16*, *StGRF17*, and *StGRF18*. In addition, an ABRE was present in all *StGRF* genes except for *StGRF2*, *StGRF3*, *StGRF4*, and *StGRF14*. These results indicate that the *StGRF* genes may have functions in response to hormones. In addition, four types of cis-elements (total of five elements) associated with responses to external environmental stresses were found: the anaerobic induction element (ARE), low-temperature responsive element (LTR), drought-responsive element (MBS), and defense-and stress-responsive elements (TC-rich repeats and W-box). All the *StGRF* genes harbored at least two types of stress-related cis-elements, except for *StGRF10*, *StGRF11*, *StGRF12*, and *StGRF13*, indicating that *StGRF* genes may be responsive to multiple environmental stresses. The different types and numbers of cis-elements in the *StGRF* gene promoters indicate that the these genes participate in different regulatory pathways during plant growth, development, and response to stress.

**Table 3:** Numbers of hormone-related and stress-related cis-elements in the upstream 2 kb regions of *StGRF* genes

cis-elements	Gene name																	
	StGRF1	StGRF2	StGRF3	StGRF4	StGRF5	StGRF6	StGRF7	StGRF8	StGRF9	StGRF10	StGRF11	StGRF12	StGRF13	StGRF14	StGRF15	StGRF16	StGRF17	StGRF18
Hormone-related cis-elements																		
ABRE	2	0	0	0	1	1	1	1	1	5	2	4	4	0	4	4	3	3
AuxRR core	0	0	0	0	0	0	0	0	0	0	0	0	0	0	0	0	1	1
TGA-element	1	1	1	1	0	0	2	2	0	0	0	1	0	0	0	0	0	0
TGACG-motif	2	2	2	2	1	1	0	0	0	1	1	3	0	0	2	2	0	0
CGTCA-motif	2	2	2	2	1	1	0	0	0	1	1	3	0	0	2	2	0	0
ERE	1	0	0	0	0	0	0	0	3	5	0	0	1	0	2	2	4	4
P-box	1	0	0	0	0	0	0	0	0	0	0	1	1	0	0	0	0	0
TATC-box	1	0	0	0	0	0	0	0	0	0	0	1	1	0	0	0	0	0
TCA-element	0	0	0	0	3	3	1	1	0	0	0	0	0	0	0	0	2	2
Stress-related cis-elements																		
ARE	1	2	2	2	5	5	1	1	2	0	0	2	0	2	1	1	3	3
LTR	0	0	0	0	0	0	0	0	0	0	0	0	0	2	0	0	0	0
MBS	1	0	0	0	1	1	0	0	1	0	3	0	0	0	0	0	0	0
TC-rich repeats	0	1	1	1	2	2	1	1	0	0	0	0	5	1	0	0	1	1
W-box	3	0	0	0	1	1	0	0	0	1	0	0	0	1	2	2	0	0

### Chromosome distribution and synteny analysis of *14–3-3* family genes in potato

**Fig. 4:**
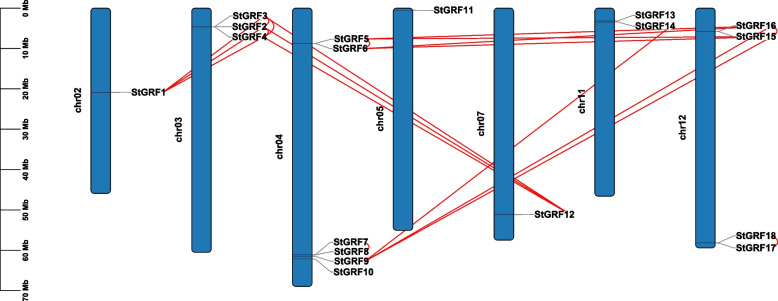
Analysis of the chromosomal distributions and duplication of the *14–3-3* genes in the potato genome. The chromosome numbers are indicated in the middle of each chromosome. Genes derived from tandem duplication and segmental duplication are connected by red lines

To ascertain the distribution of *14–3-3* genes on potato chromosomes, the positions of the *14–3-3* gene family members in the potato genome were analyzed. The 18 *StGRF* genes were distributed on 7 of the 12 chromosomes (Fig. 4), and most were located on the two ends of the chromosomes. They were most densely distributed on chromosomes 3, 4, and 12, each with three or more *StGRF* genes, and there were two genes on chromosome 11. There was only one *StGRF* gene on each of the other three chromosomes. Genome duplication events have occurred throughout the evolution of plant genomes. Gene duplication in plants mainly occurs through tandem duplication and segmental duplication. To better understand the evolution of the *StGRF* genes, we identified the genome duplication events in this gene family. We found 12 segmental duplication events and 7 tandem duplication events among the *StGRF* gene pairs. Chromosomes 4 and 12 had the most duplication events, while chromosome 11 had only one duplication event. These results indicated that some *StGRF* genes were possibly generated by gene duplication and segmental duplication events, which might be a major driving force behind *StGRF* evolution.

**Fig. 5:**
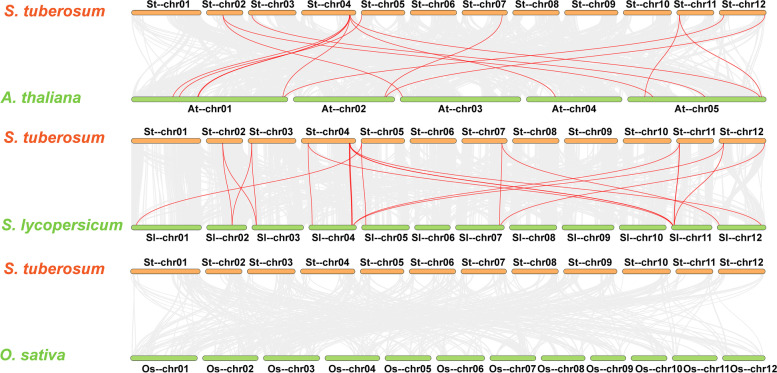
Synteny analysis of *14–3-3* genes between potato and three representative plant species. Gray lines in the background indicate the collinear blocks within potato and other plant genomes, while the red lines highlight the syntenic *StGRF* gene pairs. The prefixes St, Sl, At, and Os indicate *Solanum tuberosum*, *Solanum lycopersicum*, *Arabidopsis thaliana*, and *Oryza sativa*, respectively

To further infer the phylogenetic mechanisms of the potato *14–3-3* gene family, we constructed three comparative syntenic maps of potatoes associated with three representative species, including two dicots (*Arabidopsis* and tomato) and one monocot (rice) (Fig. 5). A total of 18 *StGRF* genes showed a syntenic relationship with genes in tomato, and 14 showed a syntenic relationship with genes in Arabidopsis, indicating that these orthologous pairs may have already existed before divergence of the ancestral lineages. Interestingly, some collinear *StGRF* gene pairs identified between potato and tomato/Arabidopsis were not found between potato and rice. Namely, there are 0 *StGRF* genes showing syntenic relationships, which may indicate that these orthologous pairs formed after the divergence of dicotyledonous and monocotyledonous plants.

### Expression profiles of *StGRF* genes in different tissues

**Fig. 6:**
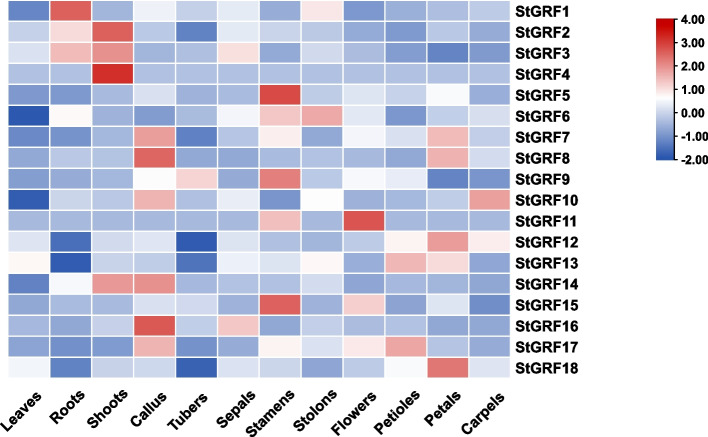
Expression profiles of *StGRF* genes in different tissues. The cluster map of the expression levels of *StGRF* genes in different tissues was generated by TBtools. The color gradient (red/white/blue) indicates the gene expression level (high to low)

To examine the possible roles of *StGRF* genes in the growth and development of potato, the expression profiles of the 18 *StGRF* genes in different tissues and organs of potato were analyzed using available RNA-seq data from the PGSC. The *StGRF* gene family members were expressed in different tissues (Fig. 6), suggesting they have diverse functions. Some *StGRF* genes were only expressed in specific tissues; for example, *StGRF4* was expressed explicitly in shoots, but the expression level was low (Additional file 3; Table S3). *StGRF1*, *StGRF2*, and *StGRF3* were highly expressed in the roots and shoots, while *StGRF11* and *StGRF15* were highly expressed in stamens and flowers, indicating that *StGRF11* and *StGRF15* might play important roles in flowering. Notably, all *StGRF* genes exhibited much lower transcript abundance in leaves. In addition, all *StGRF* genes except *StGRF6* showed low expression in tubers and stolons, suggesting that *14–3-3* genes have little effect on potato tuber formation.

### Expression patterns of *StGRF* genes under abiotic stress

**Fig. 7:**
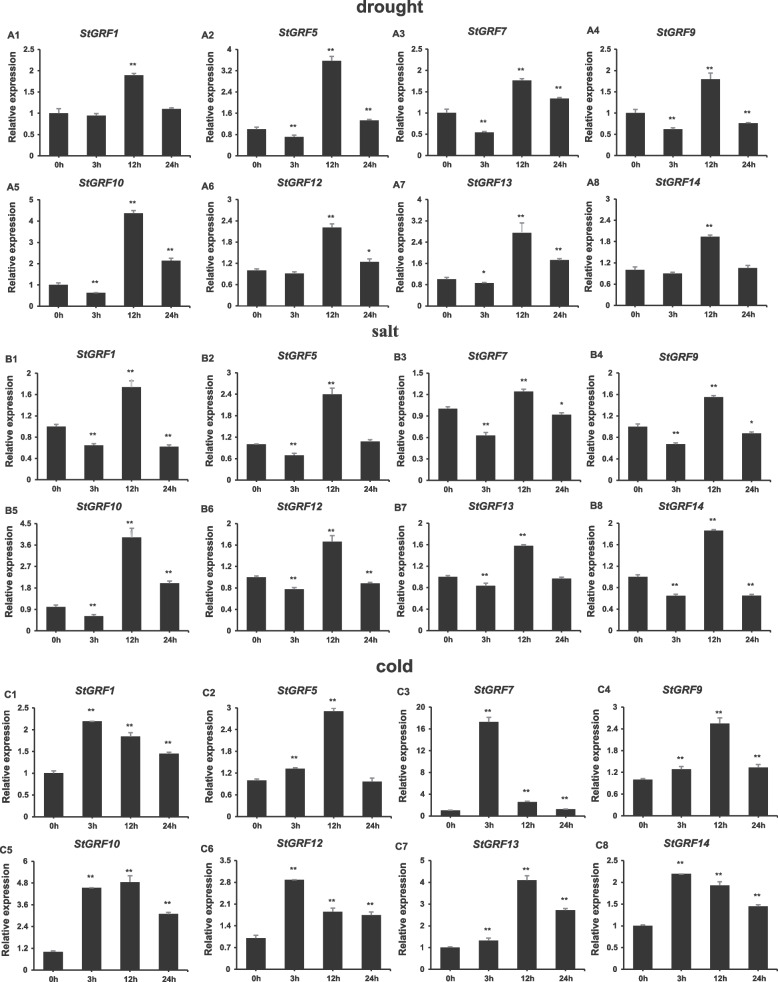
qRT-PCR expression analysis of eight selected *StGRF* genes in response to drought, salt, and cold stress. The transcript levels were analyzed with qRT-PCR using the total RNA extracted from leaves at four time points (0, 3, 12, and 24 h) (A1-A8). Treatment with 300 mM mannitol, simulating drought stress. (B1-B8). NaCl (250 mM mM) treatment. (C1-C8). Cold stress (1 °C). Asterisks on top of the bars indicate statistically significant differences between the stress and counterpart controls (*P < 0:05, **P < 0:01). Error bars represent the SD of biological replicates.

Drought, salinity, and cold are major factors affecting the production of potatoes under natural conditions. To determine whether *StGRF* genes are responsive to abiotic stress, we randomly selected eight *StGRF* genes (*StGRF1*/*5*/*7*/*9*/*10*/*12*/*13*/*14*) to further explore their expression patterns under abiotic stresses using qRT-PCR. During drought treatment, five genes (*StGRF5*/*7*/*9*/*10*/*13*) were obviously down-regulated at 3 h compared with 0 h but upregulated at 12 h. In addition, the expression of *StGRF1*, *StGRF12*, and *StGRF14* increased significantly at 12 h, then decreased at 24 h (Fig. 7, A1-A8). Under salt stress, the expression levels of all selected *StGRF* genes were upregulated considerably at the 3 h time point, then the expression levels decreased (Fig. 7, B1-B8). The changes in *StGRF1*/*7*/*12*/*14* in response to cold treatment were essentially identical, with expression peaking at the first time point (3 h) but decreasing at 12 h and 24 h. The expression of the remaining four genes (*StGRF5*/*9*/*10*/*13*) was significantly induced, peaking at 12 h, followed by an obvious decrease at 24 h (Fig. 7, C1-C8).

## Discussion

14–3-3 proteins play important roles in plant growth, development, and stress responses. The completion of genome sequencing of model plants and various crops has allowed a large number of *14–3-3* genes to be identified in several plants, including *Arabidopsis* (15) [12], tomato (12) [30], *Vitis vinifera* (grape) (11) [31], peanut (14) [32], mango (16) [33], soybean (22) [34], rice (17) [25, 35], *Hevea brasiliensis* (10) [36], cucumber (10) [37], *Medicago truncatula* (10) [38], apple (18) [39], and cassava (15) [40]. The genome sequence of potatoes was released in 2011 (PGSC, 2011). Although the *14–3-3* gene family has been well studied in several crops, there is little relevant information about the *14–3-3* gene family in potatoes [41]. In this study, a total of 18 *14–3-3* family genes (*StGRF1–18*) were identified in the potato genome using a bioinformatic method (Table 1). In marked contrast to the findings of previous studies in *Arabidopsis* [12], rice [25], and apple [39], the *St14–3-3* family genes were not evenly distributed across chromosomes. Analysis of the physicochemical properties of the St14–3-3 proteins showed that the proteins were acidic and thus stable, similar to tomato 14–3-3 proteins [30]. Secondary structure analysis showed that StGRF14 had the largest proportion of β-turns and StGRF17 had the lowest proportion; we speculate that StGRF14 may have more changes in the direction of polypeptide chains and a more complex structure than StGRF17. The analysis of the physicochemical properties of St14–3-3 proteins provides a theoretical basis for the subsequent study of their functions.

Phylogenetic analysis revealed that the potato 14–3-3 proteins could be divided into the ε and non-ε groups, which is in agreement with the results of previous studies [34, 39, 42]. The St14–3-3 proteins were most closely related to tomato 14–3-3 proteins, which is consistent with the fact that potato and tomato are *Solanaceae* plants (Fig. 2B). Analysis of intron-exon structure and conserved motifs indicated strong evolutionary conservation between proteins in each group. For example, the *StGRF* genes in the ε group had six introns, while those in the non-ε group had fewer introns (0–3; Fig. 1 and Table 1). Conserved motif analysis revealed ten conserved motifs in the ε group and non-ε group *StGRF* genes. The C-terminal motifs were highly variable; these motifs are core 14–3-3 protein structures that bind to many ligands [43], and variations in these motifs directly affect interactions between 14 and 3-3 proteins and other proteins. In addition, sequence alignment revealed that representative evolutionarily conserved signatures, such as RNL [L/V] SV [G/A] YKNV, YKDSTLIMQ LLRDNLTLWTS, and the nine α-helices, were present in all the StGRF proteins, which is similar to what has been reported for 14–3-3 proteins in other plants, such as soybean [34], cucumber [37], and grape [31].

Tandem and segmental duplication events contribute to the evolution and amplification of gene families. Tandem duplication usually refers to a gene cluster consisting of multiple family members in the same intergenic region. The most common segmental duplication event in plants produces additional family members on different chromosomes [44]. A previous study revealed that *14–3-3* genes had undergone more duplication events, particularly segmental duplication events, in dicots than in monocots [45]. In this study, the number of *14–3-3* genes in the ε group from monocots (rice) was observably smaller than that in the ε group from dicots (potato, tomato, and *Arabidopsis*) (Fig. 2C), suggesting that the *14–3-3* gene family might have evolved relatively slowly in monocots. In addition, the 18 *StGRF* genes were unevenly distributed on the 12 potato chromosomes. Fifteen *StGRF* genes arising from 12 segmental duplication events and 7 tandem duplication events were identified (Fig. 4). Therefore, segmental duplication seems to have played a primary role in the expansion of the *14–3-3* gene family in potatoes, just as in other dicots. We also identified some orthologous *14–3-3* gene pairs between potato and tomato (18) and potato and *Arabidopsis* (14) (Fig. 5), suggesting that these orthologous *14–3-3* gene pairs might have a common ancestor, and therefore might have similar functions.

Gene expression patterns can provide important evidence for understanding gene functions, and the *14–3-3* genes have been reported to have different expression patterns in various tissues of many plants [46]. In this study, most *StGRF* genes exhibited broad expression in the tested tissues (Fig. 6), indicating their vital roles in regulating various biological processes in potatoes. Similar broad expression patterns have been reported for *14–3-3* genes in many other plants, such as grape [31], cucumber [37], and mango [33]. Notably, we found that several *StGRF* genes showed particularly high expression in specific tissues, such as the shoots (*StGRF1*, *StGRF2*, *StGRF3*, and *StGRF4*), roots (*StGRF1*, *StGRF2*, and *StGRF3*), and flowers (*StGRF11* and *StGRF15*). Tissue-specific expression patterns giving insight into gene functions have also been observed for *14–3-3* genes in other plants. In *Arabidopsis*, *AtGRF12* was highly expressed in flowers and floral organs [6], and *PvGRFr* might be involved in flower development in switchgrass based on its expression pattern [47]. *PvGF14d*, *PvGF14g*, and *PvGF14q* displayed the highest transcript abundance in the flower buds of common beans [48]. In apples, several *14–3-3* genes showed particularly high expression during the floral transition stage and might participate in the floral transition through interaction with *MdTFL1* and *MdFT* [39]. Previous reports have also demonstrated the accumulation of *14–3-3* gene transcripts during fruit ripening in plants such as bananas [49], indicating their possible roles in this process. In the present study, *StGRF* genes showed lower expression in tubers and stolons, indicating that *14–3-3* genes have little effect on potato tuber formation. Taken together, the results of expression analysis suggest that the *14–3-3* gene family has various functions in plant development.

There is increasing evidence that plant *14–3-3* genes act as signal mediators in hormone signal transduction pathways regulating plant development and tolerance to various abiotic stresses [13, 20, 23, 40, 42]. For example, studies on barley *Hv14–3-3* have shown that 14–3-3 proteins are induced by ABA and participate in ABA signaling pathways [50]. By regulating the subcellular localization of the REPRESSION OF SHOOT GROWTH transcription factors, tobacco 14–3-3 proteins negatively regulate GA expression [51]. In *Arabidopsis*, 14–3-3 proteins can maintain ethylene levels by increasing the stability of 1-aminocyclopropane-1-carboxylate synthase proteins and reducing E3 ubiquitin ligase binding [52]. In addition, *Arabidopsis* ε class 14–3-3 proteins participate in plant developmental processes regulated by IAA [53]. Consistent with a similar function for *GRF* genes as mediators of hormone signal transduction pathways in potatoes, hormone-and stress-related cis-elements were found in the promoters of *StGRF* genes (Table 3). Six types of hormone-related cis-elements were identified, namely cis-elements related to ABA, auxin, MeJA, gibberellin, ethylene, and SA. qRT-PCR results revealed that the selected *StGRF* genes were differentially expressed under drought, salt, and cold stress (Fig. 7), which indicated that *StGRF* genes are regulated by different regulatory mechanisms under different stresses. Therefore, the *StGRF* genes may play specific roles in regulating plant responses to various abiotic stresses. In summary, the transcription of eight selected *StGRF* genes was induced under drought and salt stress, peaking at 12 h and then decreasing at 24 h. In addition, we also found that all the selected genes except for *StGRF5* were upregulated at all time points under cold stress, indicating that *StGRFs* play an important role in cold stress response. In previous studies, overexpression of both *14–3-3* ε and ω genes resulted in more cold-tolerant *Arabidopsis* plants with higher levels of stress-responsive proteins [54]. Apple *MdGRF11* acts as a positive regulator of tolerance to salt and drought stress by upregulating ROS-scavenging and stress-related genes [27]. Under drought stress, overexpression of *14–3GF*, which encodes a 14–3-3 protein, in maize promotes maize symbiosis and resistance to stress from arbuscular mycorrhizae. Gene expression analysis has shown that *ZmGF14–6* of maize (which encodes a 14–3-3 protein) is upregulated in response to fungal infection and salt treatment, but it is downregulated in response to drought stress [18, 26]. Thus, there is evidence that *14–3-3* genes can be induced by multiple abiotic stresses. The multiple abiotic stress responses of *StGRF* genes reflect an interconnected mechanism for inducing *StGRF* gene expression.

## Conclusion

In this study, we carried out a comprehensive genome-wide analysis of the *14–3-3* family genes in potatoes. We analyzed gene structure, conserved motifs, phylogenetic relationships, chromosomal localizations, gene duplication, promoter cis-elements, and expression profiles. Phylogenetic analysis showed that 12 StGRFs (StGRF1/2/3/4/5/6/9/10/13/14/15/16) belong to the non-ε group, and the other 6 (StGRF7/8/11/12/17/18) belong to the ε group. A total of 12 segmental duplication events and 7 tandem duplication events were identified, suggesting that segmental duplication contributed to the expansion of the *14–3-3* gene family in potatoes. Syntenic relationships between some *14–3-3* genes from potato, *Arabidopsis,* and tomato suggest that they evolved from a common ancestor. RNA-seq data showed that the *St14–3-3* genes had variable expression profiles in various tissues. In addition, the expression levels changed over time during stress, showing that *StGRFs* are involved in dynamic processes mediating in the response to multiple abiotic stress (drought, salt, and cold). In particular, seven out of eight genes examined were upregulated under cold stress treatment, indicating that *StGRF* genes play an important role in cold stress response. Our laboratory focuses on the response of potatoes to abiotic stresses, such as cold resistance, drought resistance, and salt tolerance. The *StGRF* genes have not been reported in potatoes, and these results suggest the potential roles of *StGRF* genes in the growth, development, and multiple abiotic stress responses of potatoes. These results will provide a scientific reference for the further study of *StGRF* gene functions in potatoes.

## Methods

### Plant materials and growth conditions

The tetraploid *S. tuberosum* potato cultivar ‘Desiree’ (B7) was used in this study. All of the plant materials were cultured in 30 mL Murashige and Skoog (MS) medium containing 3% sucrose and 0.8% agar at pH 5.8–6.0. The plant materials were maintained in an artificial climate chamber with a 16 h light/8 h dark photoperiod, 2500 Lx light intensity, 80% humidity, and a temperature of 22 ± 1 °C. Tissue-cultured seedlings at 3–4 weeks of age were transplanted into 10 cm × 10 cm plastic pots containing seedling substrate and grown in a solar greenhouse (14 h light/10 h dark) for 30 days before being subjected to abiotic stress. For cold stress, the plantlets were exposed to 1 °C; for salt stress, the plantlets were incubated with 250 mM NaCl; and for drought stress, the plantlets were treated with 300 mM mannitol. The aboveground portion of the plant was collected at 0, 3, 12, and 24 h after stress treatment. The collected samples were frozen in liquid nitrogen and stored at − 80 °C before RNA extraction.

### Identification of the 14–3-3 protein family members in the potato genome

All potato protein sequences were downloaded from the Potato Genome Sequencing Consortium (PGSC, http://potato.plantbiology.msu.edu/integrated_searches.shtml). To identify potato 14–3-3 protein candidates, the Hidden Markov Model (HMM) of the 14–3-3 protein domain (PF00244) was downloaded from the Sanger database (http://pfam.xfam.org/family/) and used as the query (*P* < 0.001) to search the potato protein sequence data using the HMMER 3.0 software [55]. To avoid missing probable 14–3-3 protein members, a BLASTP algorithm-based search using *Arabidopsis* 14–3-3 protein amino acid sequences as queries was conducted using an e-value ≤1e-3 as a cut-off. After removing all of the redundant sequences, the putative 14–3-3 protein sequences were submitted to CDD (https://www.ncbi.nlm.nih.gov/Structure/bwrpsb/bwrpsb.cgi), Pfam, and SMART (http://smart.embl-heidelberg.de/) to verify the integrity of the 14–3-3 protein domain (PF00244). Finally, all candidate non-redundant and high-confidence genes were designated as *S. tuberosum 14–3-3* (*St14–3-3*). These *St14–3-3* genes were named based on their positions on pseudomolecules.

### Structural characterization and sequence analysis of the *St14–3-3* genes and proteins

The chromosomal locations and intron numbers of the *St14–3-3* genes were acquired through the PGSC. The theoretical molecular weight (MW), grand average of hydropathicity (GRAVY), and isoelectric point (pI) of the deduced potato 14–3-3 proteins were determined using the ProtParam program (http://web.expasy.org/protparam). Prediction of subcellular localization was carried out with CELLO v.2.5 (http://cello.life. nctu.edu.tw/). The exon-intron structures were identified using the Gene Structure Display Server (GSDS, http://gsds.cbi.pku.edu.cn/). The MEME program (version 4.11.2, http://alternate.meme-suite.org/tools/meme) was used to identify conserved motifs in the St14–3-3 sequences, with the following parameters: any number of repetitions, maximum of 10 misfits, and an optimum motif width of 6–200 amino acid residues. The secondary structures were analyzed with SOPMA software. Phyre^2^ (http://www.sbg.bio.ic.ac.uk/phyre2/html/page.cgi?id=index) was used to obtain models of the three-dimensional protein structures.

### Sequence alignment and phylogenetic analysis

The full-length amino acid sequences of *Arabidopsis*, rice, and tomato 14–3-3 proteins were downloaded from the National Center for Biotechnology Information database (https://www.ncbi.nlm.nih.gov/). The amino acid conservation of the protein sequences was analyzed with DNAMAN software. All acquired sequences were first aligned using ClustalX (version 1.83) software with the default parameters. An unrooted neighbor-joining phylogenetic tree was constructed using MEGA6 software with 1000 bootstrap replicates.

### Analysis of cis-acting elements in *St14–3-3* gene promoters

For promoter region analysis, the 2.0 kb regions of genomic DNA upstream of the ATG start codons corresponding to *14–3-3* genes were retrieved from the potato genome database using TBtools [56]. The putative promoter region of each *14–3-3* gene was then submitted to Plant CARE (http://bioinformatics.psb.ugent.be/webtools/plantcare/html/) to identify two types of regulatory elements: hormone- and stress-related cis-elements.

### Chromosomal localization and gene duplication

The chromosomal positions of the *St14–3-3* genes were acquired from the potato genome browser at the PGSC. *St14–3-3* genes were considered duplicates if the following two criteria were met: (a) the length of the shorter aligned sequence covered > 70% of the longer sequence, and (b) the similarity of the two aligned sequences was > 70% [57, 58]. Genes on the same chromosome were considered tandem duplicate genes, and genes located on different chromosomes were considered segmental duplicate genes. Chromosomal location was plotted using TBtools software [59]. Multiple Collinearity Scan toolkit (MCScanX) was adopted to analyze the gene duplication events with the default parameters [60]. To visualize the synteny relationship of the orthologous *14–3-3* genes obtained from potatoes and other selected species, syntenic analysis maps were constructed using the Dual Synteny Plotter software [61].

### Expression analysis of potato *14–3-3* genes using RNA-seq data

Illumina RNA-seq data were downloaded from the PGSC to study the expression patterns of *St14–3-3* genes. The raw data were generated from 12 different tissues: roots, leaves, shoots, callus, tubers, sepals, stamens, stolons, flowers, petioles, petals, and carpels harvested at various developmental stages. We retrieved the fragments per kilobase per million reads (FPKM) values representing the expression levels of *St14–3-3* genes, and heat maps were generated using TBtools software [61].

### Total RNA extraction and expression analysis of *St14–3-3* genes

Primer3 (https://bioinfo.ut.ee/primer3-0.4.0/) was used to design primers specific to the *St14–3-3* genes (Additional file 1). Total RNA was extracted using the Plant Polysaccharide Polyphenol RNA Extraction Kit (GeneBetter, Beijing, China) according to the manufacturer’s instructions, and cDNA was synthesized using the Script III RT Kit with gDNA Eraser (GeneBetter, Beijing, China) as directed by the manufacturer. The *EF-1α* gene was used as the internal control for normalizing gene expression in real-time quantitative reverse transcription PCR (qRT-PCR) analysis [62]. Before the qRT-PCR analysis, 1 μL cDNA was diluted with 4 μL nuclease-free water. qRT-PCR was performed with SYBR Green Master Mix (Mei5bio, China) on a Roche Light Cycler PCR instrument (Roche, USA) using the following amplification procedure: 95 °C pre-denaturation for 5 min, followed by 40 cycles of denaturation at 95 °C for 15 s, annealing at 58 °C for 15 s, and extension at 72 °C for 45 s [63, 64]. The 2^-ΔΔCt^ method was used to analyze the expression data [65]. For each sample, three biological repeats, with three technical replicates each, were performed to assess the reliability of the results. The results were presented as means ± standard deviation (SD). All statistical analyses were conducted using a one-way analysis of variance with Tukey’s honestly significant difference test, and different letters were used to denote significant differences (*P* < 0.05).

## Supplementary Information

**Additional file 1.** Sequences of the primers use in this study.

**Additional file 2.** The similarities among StGRF proteins of potato.

**Additional file 3.** FPKM value of 14-3-3 genes that were used in this study.

## Data Availability

The data included in this article and the additional files are available. The potato reference genome assembly (DM 1–3 516 R44 v6.1) and genome annotation are available at the Spud DB website (http://spuddb.uga.edu/dm_v6_1_download.shtml). The 14–3-3 domain HMM (Hidden Markov Model) profile numbered PF00244 was extracted from the Pfam database (http://pfam.xfam.org/family/PF00244). For Desiree St14–3-3 RNA-seq data, we used DM_1–3_516_R44_potato.v6.1.TPM_gene_expression_matrix (http://spuddb.uga.edu/dm_v6_1_download.shtml). The *Arabidopsis*, rice, and tomato 14–3-3 protein sequences were downloaded from the *Arabidopsis* Information Resource (TAIR) (https://www.arabidopsis.org), Rice Genome Annotation Project (http://rice.plantbiology.msu.edu), and Solanaceae Genomics Network (https://solgenomics.net), respectively. The collection of potato materials was permitted and complied with relevant institutional, national, and international guidelines and legislation.

## Abbreviations

ABA: Abscisic Acid
GRF: G eneral regulatory factor
CDS: Coding sequence
Chr: Chromosome
AA: Amino acid
MW: Molecular weight
GRAVY: Grand average of hydropathicity
pI: Isoelectric point
ABRE: ABA-responsive element
MeJA: Methyl Jasmonate
ERE: Ethylene responsive element
SA: Salicylic acid
GA: Gibberellic acid
LTR: Low-temperature responsive element
MBS: MYB binding site
qRT-PCR: Quantitative reverse transcription PCR
MS: Murashige and Skoog
HMM: Hidden Markov Model
PGSC: Potato Genome Sequencing Consortium
GSDS: Gene Structure Display Server
MCScanX: Multiple Collinearity Scan toolkit
FPKM: Fragments per kilobase per million.
